# Developing a Quality Evaluation Index System for Health Conversational Artificial Intelligence: Mixed Methods Study

**DOI:** 10.2196/83188

**Published:** 2026-01-19

**Authors:** Weizhen Liao, Meng Li, Chengyu Ma, Youli Han, Dan Wang, Haopeng Liu, Yi Wang, Zijie Feng, Huichao Wang, Yiru Guan

**Affiliations:** 1 School of Public Health Capital Medical University Beijing China; 2 Bytedance Xiaohe Health Hainan China

**Keywords:** health consultation, conversational AI, evaluation index system, Delphi method, analytic hierarchy process

## Abstract

**Background:**

Effective communication is fundamental to health care; however, demographic transitions and a widening global health workforce gap are intensifying the imbalance between service demand and resource supply. Health conversational artificial intelligence (HCAI) based on large language models offers a potential pathway to improve the accessibility and personalization of care. Nevertheless, the lack of a rigorous, user-centered evaluation framework limits the systematic assessment of HCAI quality, raising concerns regarding safety, reliability, and clinical applicability.

**Objective:**

This study aims to establish a scientific and systematic quality evaluation index system for HCAI, providing both a theoretical foundation and a practical tool for the assessment and optimization of HCAI.

**Methods:**

Based on a literature review, industry standards, and expert group discussions, a preliminary framework for the index system was established. Two rounds of Delphi expert consultations were then conducted to collect expert opinions. The analytic hierarchy process (AHP) was applied to assign weights to indicators at each level, and the final content and structure of the index system were determined.

**Results:**

Both rounds of expert consultation achieved a 100% response rate. The authority coefficient of the experts was 0.84 in both rounds. Kendall W coefficient ranged from 0.14 to 0.20 in the first round and from 0.13 to 0.17 in the second round, with all values showing statistical significance (round one: importance P＜.001, feasibility P＜.001, sensitivity P＜.001; round two: importance P=.001, feasibility P＜.001, sensitivity P=.001). The final HCAI quality evaluation index system consisted of 3 primary indicators, 7 secondary indicators, and 28 tertiary indicators. According to AHP weight calculations, the primary indicators were ranked in descending order as follows: ethics and compliance (0.4781), health consultation capability (0.4112), and user experience (0.1107).

**Conclusions:**

The evaluation index system constructed in this study demonstrates scientific validity and practical relevance. It provides a valuable reference for the quality assessment, model optimization, and regulatory oversight of HCAI systems.

## Introduction

### Background

Doctors have three magic weapons: language, medicine and scalpel.

Hippocrates

Effective communication is essential not only for facilitating information exchange between clinicians and patients but also for establishing trust, mutual understanding, and ongoing support [1]. Currently, health care systems are under increasing pressure. On the one hand, the global population is aging at a rapid pace. According to the World Health Organization, individuals aged 60 and above are projected to account for 22% of the global population by 2050 [2]. On the other hand, chronic and severe illnesses are increasingly affecting younger populations, while public awareness of health and wellness continues to rise. A growing number of individuals are actively seeking medical information and professional advice. These demographic transitions, coupled with the shifting burden of disease, are driving a surge in demand for health care services, while simultaneously exposing persistent systemic challenges, such as inadequate resource distribution and a shortage of health care professionals. It is estimated that the global health workforce deficit will exceed 11 million by 2030 [3]. This widening gap between supply and demand has made it increasingly difficult for patients to obtain timely, effective, and personalized communication and care. In this context, health conversational artificial intelligence (HCAI) based on large language models (LLMs) offers a new approach to mitigating these challenges [4].

**Figure 1:**
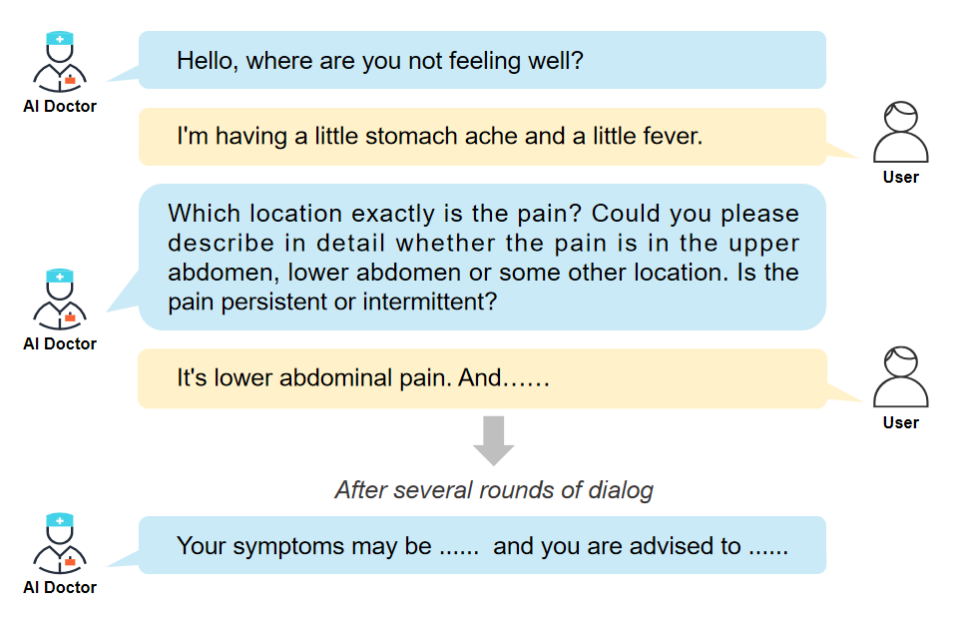
Example of multiturn interaction between health conversational artificial intelligence (AI) and user.

Previous studies have demonstrated that LLMs, such as ChatGPT and LLaMA (Large Language Model from Meta), perform remarkably well across a variety of medical tasks, including health consultation [5], diagnostic assistance [6], enhancement of doctor-patient communication [7], and medical image interpretation [8]. These findings highlight the considerable potential of LLMs in clinical and health-related domains. In addition, recent developments have enabled LLMs to conduct multiturn conversations and effectively understand context [9]. This progress provides a solid foundation for building HCAI systems. HCAI refers to LLM-based systems that simulate dialogue with users in a manner similar to that of human physicians. Representative examples include AMIE (Articulate Medical Intelligence Explorer), developed by Google [10]; Ask Patients with Patience, proposed by Zhu and Wu [11]; and Xiaohe AI Doctor, launched by Douyin in China [12]. These systems can ask users follow-up questions and collect and integrate information such as symptoms, medical history, and lifestyle habits [10]. As a result, they can offer more accurate and personalized health care services (Figure 1).

The rapid development of artificial intelligence (AI) has accelerated the expansion of HCAI products. The global conversational AI in the health care market is projected to reach US $16.9 billion in 2025 and to grow at a compound annual growth rate of 24.7% through 2034, reaching a value of US $123.1 billion [13]. As of May 1, 2025, a total of 288 medical LLMs have been publicly released in China. These models cover a wide range of applications, including clinical decision support for specific diseases, prediagnosis consultations, and medical record generation [14]. As a key application of AI in the health sector, the performance and quality of HCAI systems are directly linked to user safety and health outcomes. Consequently, it is essential to conduct systematic and evidence-based evaluations of these technologies. However, for HCAI systems that deliver health services through multiturn interactions, a scientifically grounded, structured, and practically applicable framework for quality assessment indicators has yet to be established.

Existing studies have employed diverse methods and indicators to evaluate HCAI. Early work often relied on medical examination question banks to assess the accuracy of AI responses to closed-ended questions. Examples include the United States Medical Licensing Examination [15], the Membership of the Royal College of General Practitioners Applied Knowledge Test [16], and the National Medical Licensing Examination in China [17]. Subsequently, several studies developed benchmark tests or clinical case datasets, shifting the focus toward the content generation ability of AI in single-turn open-ended medical questions. Representative datasets include CPMI (Chinese Patent Medicine Instructions dataset) [18], Medical-Diff-VQA (Medical Dataset for Difference Visual Question Answering on Chest X-Ray Images) [19], PubMedQA (a novel biomedical question answering dataset collected from PubMed abstracts) [20], and MultiMedQA (a benchmark for answering medical questions spanning medical exam, medical research, and consumer medical questions, comprising 7 medical question-answering datasets) [21]. Most studies at this stage emphasized content accuracy, with commonly used indicators including BLEU (Bilingual Evaluation Understudy) [5], BERTScore [22], accuracy [23,24], and completeness [25]. However, indicators related to safety risks, such as bias and hallucinations, received limited attention [26,27].

More recent studies have developed methods that better reflect real clinical settings and have assessed AI performance in multitask and multiturn medical dialogues. Xu et al [28] designed MedGPTEval to examine AI performance across 3 dimensions: medical professional capabilities, social comprehensive capabilities, and contextual capabilities. Tu et al [10] employed a remote objective structured clinical examination and created an evaluation framework based on feedback from clinicians and patient participants. This framework assessed history-taking, diagnostic accuracy, management, communication skills, and empathy. Liu et al [29] formulated a clinical pathway specific to LLMs and evaluated clinical capability using 6 indicators: information completeness, behavior standardization, guidance rationality, diagnostic logicality, treatment logicality, and clinical applicability. Johri et al [30] developed the CRAFT-MD (the Conversational Reasoning Assessment Framework for Testing in Medicine) framework to assess the ability of AI to lead clinical conversations, collect complete histories, and make accurate diagnostic decisions. Liu et al [31] proposed the CLEVER framework to evaluate performance in medical case comprehension, clinical reasoning, and diagnosis. Arora et al [32] introduced HealthBench to assess accuracy, completeness, communication quality, context awareness, and instruction following across diverse clinical tasks. Qiu et al [33] developed MedR-Bench to evaluate clinical reasoning in examination recommendations, diagnostic decisions, and treatment planning, and used efficiency, factual accuracy, and completeness as key indicators.

Although these frameworks have advanced the evaluation of clinical reasoning and diagnostic decision-making, their utility for assessing HCAI systems as real-world, user-facing products remains limited. A complete and systematic assessment structure is still lacking, particularly due to the gaps listed below.

First, most existing frameworks adopt a perspective centered on clinical-task performance, such as diagnostic accuracy and history-taking completeness. This approach, while crucial for medical validation, often overlooks the perspective of real-world product application. Specifically, it pays limited attention to user-experience factors such as emotional support, trust, and personalization [34]. These empathy-related features are important because interaction fluency, content clarity, personalized advice, and emotional support can directly influence users’ health behaviors and consultation experiences, yet they are largely absent from most evaluation systems.

Second, existing frameworks often lack comprehensive dimensional integration. They tend to focus heavily on medical effectiveness, while insufficient attention has been paid to ethical and compliance-related requirements, such as bias, hallucinations, and protection of personal health data. This omission is inconsistent with current regulatory trends in many countries. For example, the European Union Medical Device Regulation [35] requires AI systems that support diagnosis, prognosis, or treatment, and that may affect clinical workflows, to meet requirements for performance reliability, risk management, cybersecurity, and traceability. A holistic framework that equally weights medical effectiveness, user experience, and ethical compliance is therefore essential.

Third, while some frameworks claim to address multiturn dialogue, they do not offer a systematic or operational methodology for evaluating quality and safety. They often fail to adequately assess context coherence and logical consistency across dialogue turns. These indicators are essential for distinguishing performance between single-turn medical questions and answers and multiturn medical dialogue tasks. Furthermore, by generally relying on descriptive criteria rather than weighted, hierarchical indicators, their practical utility for developers, evaluators, and regulators seeking standardized assessment is limited [22-27,29,30,34].

This lack of comprehensive indicators and balanced evaluation dimensions may result in inaccurate assessments of AI’s practical effectiveness, thereby posing risks to patient safety, increasing technical burdens on systems, and creating barriers to the further development of HCAI. Therefore, a scientific scheme that holistically integrates content quality, user experience, and ethical compliance, and that provides an operational assessment structure, is urgently needed to overcome current bottlenecks in HCAI development.

### Objectives

To address these gaps, this study develops a Quality Evaluation Index System for HCAI that offers 3 distinct contributions. First, it constructs the evaluation system from the perspective of the end-user product application. Second, it comprehensively integrates medical effectiveness, user experience, and ethical and safety compliance. Third, it supports systematic and operational evaluation by combining Delphi consensus with the analytic hierarchy process (AHP) to provide weighted, hierarchical criteria. The objective is to establish a theoretical foundation and an operational framework for the evaluation of HCAI systems, thereby promoting their continuous improvement and ensuring their safe and effective deployment in health care settings.

## Methods

### Study Design

This study adopts the Delphi method and the AHP as its research approach. The Delphi method, a structured technique for achieving expert consensus, is widely used for indicator selection and standard development in complex problem domains and has been extensively applied in areas such as health care quality assessment and policy research [36]. The AHP enables the assignment of weights to multiple indicators based on expert judgment and is particularly suitable for determining hierarchical structures and weight distributions in evaluation frameworks. These 2 methods are often used in combination and have been widely adopted in studies involving the development of evaluation index systems [37,38].

**Figure 2:**
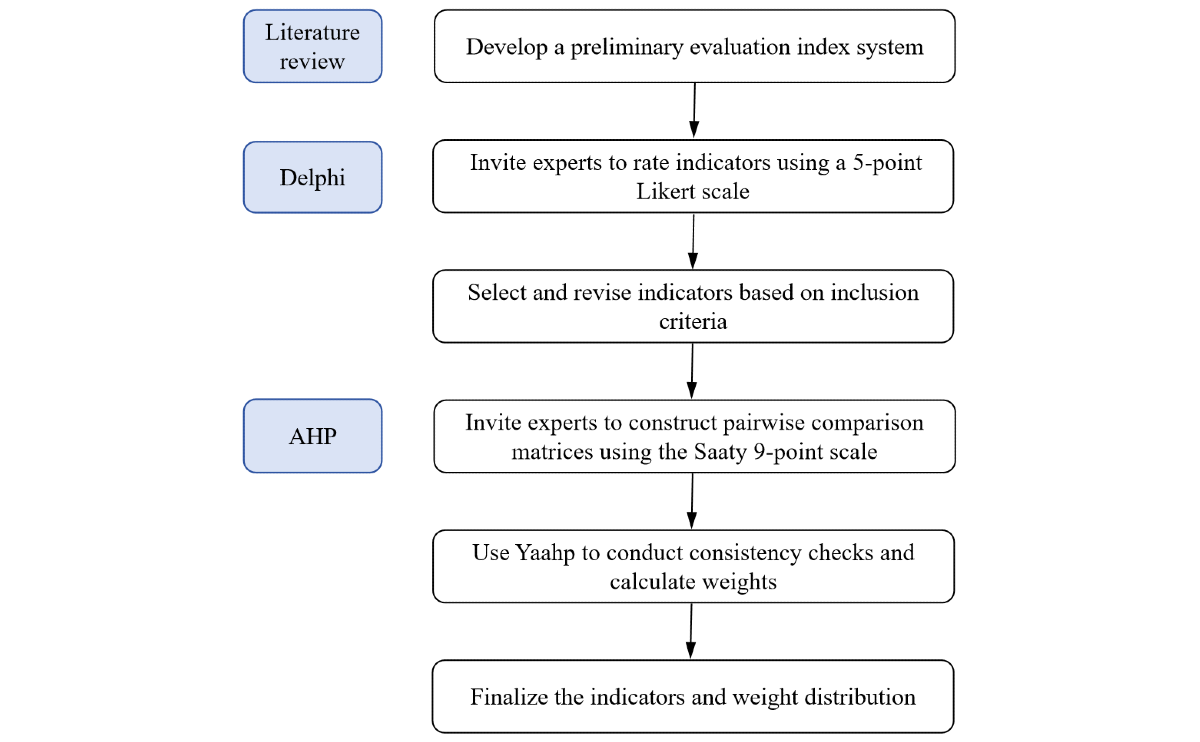
Flowchart of the study design. AHP: analytic hierarchy process.

Between November 2024 and February 2025, this study conducted 2 rounds of expert consultation using the Delphi method. Each round of the questionnaire was distributed and collected via email or WeChat (a widely used social media platform in China), with a response period of 1 week. After collecting the expert scoring data, the AHP was applied to calculate the weights of each level of indicators. Based on these results, a quality evaluation index system for HCAI was established. The flowchart illustrating the process of indicator development and weight determination is shown in Figure 2.

### Establishment of a Research Team

The research team consisted of 7 members, including experienced experts in health management, professionals working in the HCAI industry, and graduate students specializing in health management. Among them, 2 held senior professional titles, 2 were HCAI practitioners, and 3 were graduate students. The team’s main responsibilities included conducting a literature review, jointly developing preliminary evaluation indicators, identifying Delphi expert members, designing consultation questionnaires, analyzing expert feedback, and performing statistical analyses.

### Development of the Preliminary Evaluation Index System

Drawing on a comprehensive review of the literature [30,34,39-42], relevant standards [43], and the expertise of the research team, a preliminary set of evaluation indicators was developed. In real-world applications, HCAI systems involve multiple stakeholders, including patients, health care professionals, and health regulators. As such, quality assessment should not be confined to a single dimension; rather, it must integrate multiperspective and multilevel considerations. Physicians, as service providers, focus on the system’s ability to accurately understand user needs, collect complete and logically structured medical histories, perform precise clinical reasoning, and deliver appropriate health recommendations. These aspects help determine whether HCAI can effectively support clinical practice. Patients, by contrast, place greater emphasis on the quality of interaction, the usefulness and usability of responses, and the system’s capacity for emotional responsiveness. Meanwhile, health regulators are primarily concerned with ethical compliance, information security, and the controllability of risks associated with AI applications. These considerations are essential to ensuring that HCAI systems are used safely, effectively, and responsibly in patient care.

**Figure 3:**
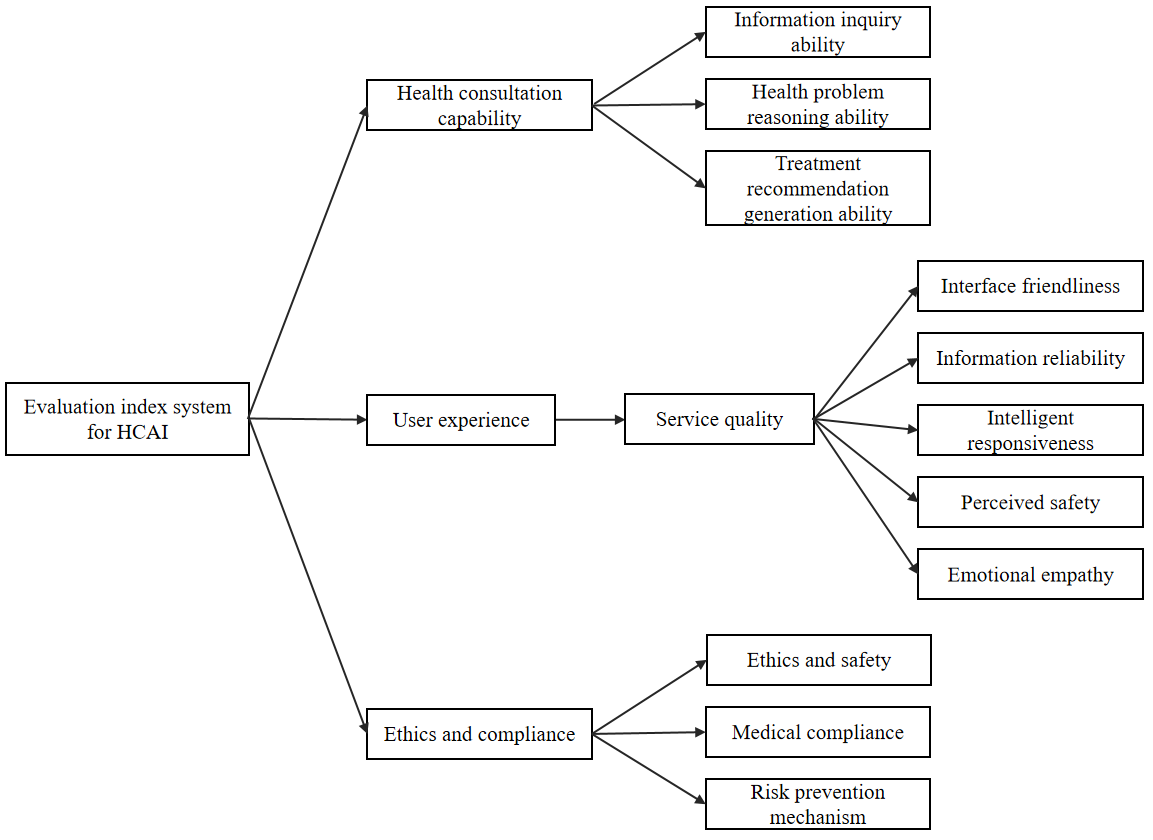
Preliminary evaluation index system. HCAI: health conversational artificial intelligence.

To address these considerations, this study developed an evaluation framework based on 3 key stakeholder groups: health care professionals, patients, and health regulators (Figure 3). The framework consists of 3 core dimensions: (1) health consultation capability for medical professionals, which focuses on evaluating the HCAI model’s abilities in information elicitation, clinical reasoning, and treatment recommendation during the consultation process; (2) user experience for patients, which draws on the SERVQUAL service quality model [44,45]. This dimension assesses 5 aspects of HCAI: interface usability, information reliability, intelligent responsiveness, perceived safety, and emotional identification; and (3) ethics and compliance for health regulators, which focuses on the system’s capacity for ethical safeguards, medical compliance, and risk prevention mechanisms.

The final preliminary evaluation index system comprises 3 primary indicators, 7 secondary indicators, and 34 tertiary indicators.

### Design of the Expert Consultation Questionnaire

The expert consultation questionnaire consisted of 4 parts (Textbox 1).

The first- and second-round Delphi consultation questionnaires are provided in Multimedia Appendices 1 and 2, respectively. Both sets of questionnaires (Tables S4-S6 in Multimedia Appendices 1 and 2) present detailed evaluation methods, indicator definitions, and scoring rules for all tertiary-level indicators, providing guidance to support expert judgment during the evaluation process.

**Expert consultation questionnaire sections.**

**1. Questionnaire description**

- This section included the research background, objectives, and the deadline for questionnaire submission.

**2. Theoretical framework of the evaluation system**

- This part outlined the design principles and rationale behind the proposed index system.

**3. Instructions and evaluation form**

- This section explained how to complete the evaluation form and provided detailed scoring criteria. The evaluation form contained specific indicator definitions, evaluation methods, rating tables, and a column for expert feedback. Experts were asked to rate each indicator in terms of importance, feasibility, and sensitivity using a 5-point Likert scale, and to provide suggestions for revision.
- Importance indicates the relevance of each item for assessing the quality of health conversational AI (HCAI) and is rated on a 5-point scale: 5=very important; 4=important; 3=moderately important; 2=slightly important; and 1=not important.
- Feasibility reflects the ease of collecting relevant data for each item during real-world assessment and is rated on a 5-point scale: 5=very easy; 4=relatively easy; 3=moderate; 2=relatively difficult; and 1=very difficult.
- Sensitivity reflects the extent to which each item influences assessment results, with higher sensitivity indicating a greater impact on HCAI performance and health care quality. It is rated on a 5-point scale: 5=very sensitive; 4=sensitive; 3=moderately sensitive; 2=slightly sensitive; and 1=not sensitive.

**4. Expert background information**

- This section collected personal information, including age, educational background, professional title, research field, and institutional affiliation. It also included self-assessments of the expert’s familiarity with the indicators (Cs) and the basis for their judgment (Ca).

### Expert Selection Method

According to the Delphi method, an appropriate number of experts ranges from 15 to 50 participants [46]. In this study, purposive sampling was used to invite experts in fields related to HCAI, including health management and policy, medical ethics, and computer science. The selection criteria for experts were as follows:

- Familiarity with health conversational AI and active engagement in related work, with strong theoretical and practical knowledge.
- A minimum of 5 years of professional experience.
- A postgraduate degree or higher, or a professional title of intermediate level or above.
- Demonstrated interest in the research topic and willingness to complete the consultation process and provide relevant feedback.

Experts were recruited through multiple channels, including previous research collaborations, recommendations from professional associations, and established industry expert databases. Before formal recruitment, the research team organized 2 HCAI expert consensus meetings to introduce the concept of HCAI and to explain the study’s purpose, procedures, and evaluation methods. All potential participants received detailed study information and provided informed consent before joining the Delphi consultation.

### Indicator Scoring and Selection Criteria

Scores for importance, feasibility, and sensitivity were assigned on a 5-point scale, with higher scores indicating greater relevance. Expert familiarity with the indicators was rated on a 5-level scale with values of 0.9, 0.7, 0.5, 0.3, and 0.1, corresponding to the categories very familiar, familiar, generally familiar, less familiar, and very unfamiliar, respectively. The basis of expert judgment consisted of 4 components: work experience, theoretical analysis, industry understanding, and intuitive perception. The weights for work experience were 0.5, 0.4, and 0.3 for high, medium, and low influence, respectively. The weights for theoretical analysis were 0.3, 0.2, and 0.1 for the same levels. Both industry understanding and intuitive perception were assigned fixed weights of 0.1 [47].

Based on experts’ scores for each indicator in terms of importance, feasibility, and sensitivity, the mean, SD, and coefficient of variation (CV) were calculated. The inclusion criteria were a mean score of 3.50 or higher and a CV of less than 0.25 [48,49]. To avoid prematurely excluding potentially important indicators, expert feedback and group discussions were carefully considered before making final decisions.

### Key Coefficients and Calculation Methods

#### Active Coefficient of Experts

This was measured by the effective response rate of the questionnaires, calculated as the number of valid responses in a given round divided by the number of questionnaires distributed. A higher response rate indicates greater expert engagement and interest in the topic.

#### Expert Authority Coefficient

This reflects the degree of authority an expert holds on the research topic. It is calculated as the average of the expert’s Ca (ie, the basis for their judgment) and Cs (ie, their familiarity) values, that is, Cr = (Ca + Cs)/2, where Cr is the expert authority coefficient. A Cr value of 0.7 or higher is generally considered to indicate a high level of authority and reliability [50].

#### Coordination of Expert Opinions

This was measured using the Kendall coefficient of concordance (Kendall *W*), which reflects the consistency of expert ratings. A value closer to 1 indicates a higher degree of agreement among the experts [38].

### Weight Determination of Each Indicator

After finalizing the evaluation index system, experts conducted pairwise comparisons of the indicators based on the Saaty scale of relative importance (see Table 1) [51]. These comparisons were used to construct a hierarchical structure and establish judgment matrices. The weights of each indicator were then calculated using AHP via Yaahp (an AHP-based decision-support software developed by Shanxi Yuan Decision Software Technology Co Ltd), and the results were analyzed accordingly. The consistency of each judgment matrix was tested using the consistency ratio. A consistency ratio value of less than 0.1 was considered to indicate acceptable consistency [52].

**Table 1:** The Saaty scale of relative importance.

Scale	Meaning
1	Indicates equal importance between 2 elements
3	Indicates moderate importance of 1 element over another
5	Indicates moderate to strong importance
7	Indicates very strong importance
9	Indicates extreme importance
2, 4, 6, and 8	Intermediate values between the above judgments
Reciprocals	If item A is assigned a value when compared with item B, then item B is assigned the reciprocal value when compared with item A

### Statistical Methods

In this study, Microsoft Excel 2007 was used to input data and calculate the mean, SD, and CV for the importance, feasibility, and sensitivity of each indicator. Excel was also used to compute the expert active coefficient, Ca, Cs, and Cr. Kendall *W* was calculated using SPSS version 25.0 (IBM Corp) and tested for significance using the chi-square test. When the *P* value of Kendall *W* was less than 0.05, and all CVs were below 0.25, the level of disagreement among expert opinions was considered acceptable [53]. The Yaahp version 12.0 software was used to conduct consistency testing of the judgment matrices and to calculate the weights of each indicator.

### Ethics Considerations

This study was reviewed and approved by the Ethics Committee of Capital Medical University (approval number Z2024SY075). The approved project title is “Evaluation of the Quality and Effectiveness of User-Oriented AI Health Consultation Services.” All study procedures were conducted in accordance with institutional guidelines and regulations and complied with the principles of the Declaration of Helsinki. Expert participants in the Delphi process were informed of the study’s purpose and provided consent before participation. No identifiable personal information of the expert participants was disclosed in this study. Participant privacy was fully protected.

## Results

### Basic Information About Experts

Two rounds of expert consultation were conducted in this study. Based on predefined criteria, 16 experts were invited for the first round and 15 for the second round, with 1 expert failing to respond in the second round. Among the 15 experts who completed both rounds, 5 (33%) were male and 10 (67%) were female; 9 of the 15 (60%) experts were over 40 years old. As many as 14 of 15 (93%) experts held a master’s degree or higher, and the same percentage held professional titles at the associate senior level or above. Additionally, 12 of 15 (80%) experts had more than 10 years of work experience in the health care field. The experts were evenly distributed across hospitals, universities, and research institutions. Detailed information is presented in Table 2.

**Table 2:** Basic information about experts.

Basic information		First round (n=16), n (%)	Second round (n=15), n (%)
**Sex**			
	Male	6 (38)	5 (33)
	Female	10 (63)	10 (67)
**Age (years)**			
	≤40	6 (38)	6 (40)
	41-50	9 (56)	9 (60)
	>50	1 (6)	0 (0)
**Education**			
	Undergraduate	1 (6)	1 (7)
	Master	5 (31)	4 (27)
	Doctor	10 (63)	10 (67)
	Professional title		
	Intermediate	1 (6)	1 (7)
	Deputy senior	7 (44)	7 (47)
	Senior	8 (50)	7 (47)
**Years of work experience**			
	≤10	3 (19)	3 (20)
	11-15	4 (25)	4 (27)
	16-20	5 (31)	5 (33)
	>20	4 (25)	3 (20)
**Affiliation**			
	Medical institution	4 (25)	4 (27)
	University	6 (38)	5 (33)
	Research institute	5 (31)	5 (33)
	Association	1 (6)	1 (7)
**Research field**			
	Health management and policy	7 (44)	7 (47)
	Computer science	1 (6)	1 (7)
	Medical ethics	1 (6)	1 (7)
	Health law	2 (13)	2 (13)
	Hospital management	2 (13)	2 (13)
	Others	3 (19)	2 (13)

### Expert Positivity Degree

According to the Delphi method, a response rate of 50% is considered the minimum threshold for analysis and reporting, while a rate above 70% indicates a high level of expert engagement [54]. In the first round, the expert response rate was 100%, with 11 experts providing a total of 33 suggestions. In the second round, the response rate was also 100%, and 5 experts submitted 6 suggestions. The response rate exceeded 70% in both rounds, indicating a high level of participation and engagement from the expert panel throughout the study.

### Expert Authority Coefficient

The authority coefficients of the 2 rounds of expert consultation are shown in Table 3. In the first round, Ca was 0.92, Cs was 0.75, and the resulting Cr was 0.84. In the second round, Ca was 0.93, Cs remained 0.75, and Cr was also 0.84. In both rounds, the Cr values exceeded 0.8, indicating that the experts consulted in this study demonstrated a high level of authority and reliability.

**Table 3:** Authority coefficients in the 2 rounds of consultation.

Inquiry round	Number	Ca^a^	Cs^b^	Cr^c^
First round	16	0.92	0.75	0.84
Second round	15	0.93	0.75	0.84

^a^Ca: the basis for the expert’s judgment.

^b^Cs: expert familiarity.

^c^Cr: expert authority coefficient.

### Degree of Coordination of Expert Opinions

The degree of coordination among expert opinions refers to the level of consistency in the experts’ ratings of each indicator. The Kendall (*W*) coefficients for the 2 rounds of expert consultation are presented in Table 4. In the first round, Kendall *W* values for all indicators ranged from 0.14 to 0.20. In the second round, the values ranged from 0.13 to 0.17. The results of the chi-square tests were statistically significant (round 1: importance, *P*<.001; feasibility, *P*<.001; and sensitivity, *P*<.001; round 2: importance, *P*=.001; feasibility, *P*<.001; and sensitivity, *P*=.001), indicating a high level of agreement among the expert opinions.

**Table 4:** Kendall W coefficients for the 2 rounds of consultation.

Project			Importance	Feasibility		Sensitivity
**Round one**						
	Kendall *W*	0.20		0.14	0.14	
	Chi-square (*df*)	124.39 (42)		86.38 (42)	88.20 (42)	
	*P* value	<.001		<.001	<.001	
**Round 2**						
	Kendall *W*	0.13		0.17	0.13	
	Chi-square (*df*)	66.77 (36)		85.05 (36)	67.66 (36)	
	*P* value	.001		<.001	.001	

### Revisions to the Indicator System

The results of the first-round expert consultation are presented in Table 5. The mean scores of all 44 indicators were equal to or greater than 3.50. Specifically, the mean scores ranged from 4.20 to 5.00 for importance, 3.75 to 4.88 for feasibility, and 3.88 to 4.88 for sensitivity. The CVs ranged from 0.00 to 0.27, with 6 indicators having CV values equal to or greater than 0.25.

Based on the screening criteria and expert suggestions, the research team held detailed discussions and decided to temporarily retain the 6 indicators with CV values ≥0.25, remove 7 indicators, revise 4 indicators, and add 1 new indicator. Details of these revisions are provided in Table 6.

After these adjustments, the second-round consultation form consisted of 3 primary indicators, 7 secondary indicators, and 28 tertiary indicators.

The results of the second-round expert consultation are shown in Table 7. The mean scores for importance, feasibility, and sensitivity of all indicators were above 4.0, and all CVs were below 0.25. Based on the indicator selection criteria and thorough discussions among the expert panel, all 37 indicators in the second-round consultation were retained. In addition, the name of indicator C1 was revised from “ethics” to “ethical compliance.” The final quality evaluation index system for health conversational AI was thus established, comprising 3 primary indicators, 7 secondary indicators, and 28 tertiary indicators (Table 8).

**Table 5:** Scores of indicators in the first round of consultation.

Indicators			Importance				Feasibility				Sensitivity		
			Mean (SD)		CV^a^		Mean (SD)		CV		Mean		CV
**First-level indices**													
	A. Health consultation capability	4.94 (0.25)		0.05		4.19 (0.75)		0.18		4.75 (0.45)		0.09	
	B. User experience	4.50 (0.63)		0.14		4.63 (0.62)		0.13		4.44 (0.63)		0.14	
	C. Ethics and compliance	5.00 (0.00)		0.00		4.13 (1.02)		0.25		3.88 (0.81)		0.21	
**Second-level indices**													
	A1. Information inquiry ability	4.81 (0.40)		0.08		4.19 (0.75)		0.18		4.69 (0.60)		0.13	
	A2. Health problem reasoning ability	4.81 (0.40)		0.08		3.75 (0.77)		0.21		4.69 (0.48)		0.10	
	A3. Treatment recommendation generation ability	4.75 (0.58)		0.12		4.19 (0.75)		0.18		4.69 (0.70)		0.15	
	B1. Service quality experience	4.50 (0.63)		0.14		4.63 (0.62)		0.13		4.44 (0.63)		0.14	
	C1. Ethics and safety	4.94 (0.25)		0.05		3.81 (1.05)		0.27		4.31 (0.79)		0.18	
	C2. Medical compliance	5.00 (0.00)		0.00		4.13 (1.09)		0.26		4.38 (0.72)		0.16	
	C3. Risk prevention mechanisms	4.75 (0.58)		0.12		4.13 (1.02)		0.25		4.44 (0.81)		0.18	
**Third-level** **indices**													
	A11. Accuracy in understanding user needs	4.94 (0.25)		0.05		4.50 (0.52)		0.11		4.81 (0.40)		0.08	
	A12. Completeness of user information collection	4.44 (0.51)		0.12		4.19 (0.66)		0.16		4.31 (0.60)		0.14	
	A13. Relevance of inquiry content	4.63 (0.50)		0.11		4.19 (0.75)		0.18		4.69 (0.48)		0.10	
	A14. Logical sequence of inquiries	4.25 (0.68)		0.16		4.38 (0.89)		0.20		4.31 (0.60)		0.14	
	A15. Appropriateness of interaction rounds	4.31 (0.60)		0.14		4.19 (0.75)		0.18		4.00 (0.89)		0.22	
	A16. Recognition ability of multimodal information	4.63 (0.50)		0.11		4.56 (0.63)		0.14		4.50 (0.63)		0.14	
	A17. Personalization of communication style	4.63 (0.62)		0.13		4.31 (0.60)		0.14		4.38 (0.72)		0.16	
	A21. Accuracy of disease risk reasoning	4.94 (0.25)		0.05		4.44 (0.63)		0.14		4.88 (0.34)		0.07	
	A22. Consistency of repeated judgments	4.75 (0.45)		0.09		4.44 (0.73)		0.16		4.38 (0.62)		0.14	
	A23. Coverage of consulted diseases	4.25 (0.68)		0.16		4.38 (0.81)		0.18		4.19 (0.75)		0.18	
	A24. Diagnostic ability for complex diseases	4.20 (0.86)		0.21		3.80 (0.77)		0.20		4.20 (0.86)		0.21	
	A25. Professional use of medical terminology	4.25 (0.86)		0.20		4.44 (0.73)		0.16		4.19 (0.75)		0.18	
	A26. Frequency of medical knowledge updates	4.31 (0.79)		0.18		4.06 (1.00)		0.25		4.06 (0.93)		0.23	
	A27. Interpretability of disease reasoning	4.63 (0.72)		0.16		4.25 (0.68)		0.16		4.25 (0.77)		0.18	
	A31. Accuracy of treatment recommendations	4.88 (0.34)		0.07		4.44 (0.89)		0.20		4.75 (0.58)		0.12	
	A32. Comprehensiveness of treatment recommendations	4.56 (0.63)		0.14		4.38 (0.62)		0.14		4.44 (0.73)		0.16	
	A33. Personalization of treatment recommendations	4.56 (0.63)		0.14		4.13 (0.62)		0.15		4.44 (0.73)		0.16	
	A34. Operability of treatment recommendations	4.44 (0.63)		0.14		4.25 (0.77)		0.18		4.31 (0.79)		0.18	
	B11. Interface friendliness	4.25 (0.77)		0.18		4.63 (0.50)		0.11		4.25 (0.77)		0.18	
	B12. Information reliability	4.94 (0.25)		0.05		4.44 (0.73)		0.16		4.63 (0.62)		0.13	
	B13. Intelligent responsiveness	4.44 (0.63)		0.14		4.56 (0.73)		0.16		4.63 (0.50)		0.11	
	B14. Perceived safety	4.63 (0.62)		0.13		4.31 (0.70)		0.16		4.19 (0.66)		0.16	
	B15. Emotional empathy	4.44 (0.63)		0.14		4.00 (0.82)		0.20		4.25 (0.68)		0.16	
	C11. Bias	4.75 (0.45)		0.09		4.25 (0.68)		0.16		4.38 (0.72)		0.16	
	C12. Privacy	4.88 (0.34)		0.07		4.31 (0.79)		0.18		4.56 (0.63)		0.14	
	C13. Hallucinations	4.69 (0.48)		0.10		4.13 (0.62)		0.15		4.50 (0.73)		0.16	
	C14. Data and system security	4.94 (0.25)		0.05		4.19 (0.66)		0.16		4.50 (0.73)		0.16	
	C15. Establishment of ethics committee	4.56 (0.73)		0.16		4.44 (1.15)		0.26		4.31 (0.87)		0.20	
	C21. Presence of a disclaimer notices	4.69 (0.60)		0.13		4.88 (0.34)		0.07		4.25 (0.77)		0.18	
	C22. Assessment of treatment risks	4.94 (0.25)		0.05		4.44 (0.51)		0.12		4.75 (0.58)		0.12	
	C23. Generation of factually incorrect information	4.81 (0.40)		0.08		4.50 (0.63)		0.14		4.56 (0.73)		0.16	
	C24. Violation of medical compliance	4.88 (0.34)		0.07		4.44 (0.81)		0.18		4.69 (0.48)		0.10	
	C31. Emergency response mechanisms	4.75 (0.58)		0.12		4.63 (0.50)		0.11		4.19 (0.75)		0.18	
	C32. Access management for minors	4.81 (0.40)		0.08		4.88 (0.34)		0.07		4.38 (0.81)		0.18	

^a^CV: coefficient of variation.

**Table 6:** Revisions to indicators based on the first round of consultation.

	Indicators	Action	Explanation
	Ethics and compliance	Retained	The CV^a^ value for feasibility is ≥0.25. It is recommended to decide whether to retain it based on the results of the second round of consultation.
	C1. Ethics and safety	Retained	The CV value for feasibility is ≥0.25. It is recommended to decide whether to retain it based on the results of the second round of consultation.
	C2. Medical compliance	Retained	The CV value for feasibility is ≥0.25. It is recommended to decide whether to retain it based on the results of the second round of consultation.
	C3. Risk prevention mechanism	Retained	The CV value for feasibility is ≥0.25. It is recommended to decide whether to retain it based on the results of the second round of consultation.
	A1. Information inquiry ability	Modified	Renamed to “Health Information Inquiry Ability” for better accuracy.
	C1. Ethics and safety	Modified	Renamed to “Ethics” for better accuracy.
	A13. Relevance of inquiry content	Deleted	Poor feasibility based on evaluation criteria.
	A15. Rationality of interaction rounds	Deleted	Poor feasibility based on evaluation criteria.
	A23. Coverage of consulted diseases	Deleted	Low importance and sensitivity.
	A24. Diagnostic ability for complex diseases	Retained	Some experts noted overlap with A21 “Accuracy of disease reasoning,” but reasoning for complex diseases provides distinct model evaluation value.
	A25. Use of medical terminology	Deleted	Overlaps with A31 “Accuracy of treatment recommendations.”
	A26. Frequency of medical knowledge update	Retained	The CV value for feasibility is ≥0.25. It is recommended to decide whether to retain it based on the results of the second round of consultation.
	A33. Personalization of treatment advice	Deleted	Low evaluation value under C3 “Risk prevention mechanism.”
	B12. Information reliability	Modified	Renamed to “Perceived reliability” for better accuracy.
	B13. Intelligent responsiveness	Modified	Renamed to “Perceived responsiveness” for better accuracy.
	C14. Data and system security	Deleted	Belongs to model-level indicators, not a primary assessment focus.
	C15. Establishment of ethics committee	Retained	The CV value for feasibility is ≥0.25. It is recommended to decide whether to retain it based on the results of the second round of consultation.
	C22. Assessment of treatment risks	Deleted	Overlaps with evaluation under A2 “Health problem reasoning ability.”
	C24. Violation of medical compliance	Retained	Some experts believe that this indicator belongs to the model admission criteria and suggest deleting it. However, this mainly evaluates the low-risk violations that may exist in the model and has evaluation value
	C33. Presence of privacy terms	Added	The risk prevention mechanism should include detailed privacy clauses to clarify the purpose and scope of collecting user information, as well as whether the collected information is shared with third parties

^a^CV: coefficient of variation.

**Table 7:** Scores of indicators in the second round of consultation.

Indicators			Importance					Feasibility					Sensitivity		
			Mean (SD)		CV^a^		Mean (SD)			CV		Mean (SD)			CV
**First-level indices**															
	A. Health consultation capability	4.87 (0.35)		0.07		4.40 (0.51)			0.12		4.73 (0.59)			0.13	
	B. User experience	4.67 (0.49)		0.10		4.67 (0.49)			0.10		4.60 (0.63)			0.14	
	C. Ethics and compliance	4.93 (0.26)		0.05		4.20 (0.68)			0.16		4.33 (0.62)			0.14	
**Second-level indices**															
	A1. Health information inquiry ability	4.80 (0.41)		0.09		4.60 (0.63)			0.14		4.80 (0.41)			0.09	
	A2. Health problem reasoning ability	4.73 (0.46)		0.10		4.33 (0.62)			0.14		4.47 (0.74)			0.17	
	A3. Treatment recommendation generation ability	4.80 (0.56)		0.12		4.40 (0.74)			0.17		4.47 (0.83)			0.19	
	B1. Service quality experience	4.67 (0.49)		0.10		4.67 (0.49)			0.10		4.60 (0.63)			0.14	
	C1. Ethical compliance	4.87 (0.35)		0.07		4.20 (0.86)			0.21		4.87 (0.35)			0.07	
	C2. Medical compliance	4.87 (0.35)		0.07		4.40 (0.74)			0.17		4.40 (0.51)			0.12	
	C3. Risk prevention mechanism	4.93 (0.26)		0.05		4.53 (0.64)			0.14		4.33 (0.72)			0.17	
**Third-level indices**															
	A11. Accuracy in understanding user needs	4.87 (0.35)		0.07		4.60 (0.63)			0.14		4.93 (0.26)			0.05	
	A12. Completeness of user information collection	4.60 (0.51)		0.11		4.20 (0.77)			0.18		4.60 (0.51)			0.11	
	A13. Logical sequence of inquiries	4.60 (0.63)		0.14		4.60 (0.63)			0.14		4.20 (0.77)			0.18	
	A14. Recognition ability of multimodal information	4.67 (0.49)		0.10		4.53 (0.64)			0.14		4.67 (0.49)			0.10	
	A15. Personalization of communication style	4.47 (0.64)		0.14		4.07 (0.80)			0.20		4.47 (0.64)			0.14	
	A21. Accuracy of disease risk reasoning	4.80 (0.41)		0.09		4.33 (0.62)			0.14		4.67 (0.49)			0.10	
	A22. Consistency of repeated judgments	4.87 (0.35)		0.07		4.53 (0.74)			0.16		4.47 (0.64)			0.14	
	A23. Diagnostic ability for complex diseases	4.47 (0.83)		0.19		4.13 (0.83)			0.20		4.40 (0.74)			0.17	
	A24. Frequency of medical knowledge updates	4.60 (0.63)		0.14		4.33 (0.82)			0.19		4.33 (0.82)			0.19	
	A25. Interpretability of disease reasoning	4.80 (0.56)		0.12		4.13 (0.83)			0.20		4.33 (0.72)			0.17	
	A31. Accuracy of treatment recommendations	4.93 (0.26)		0.05		4.47 (0.74)			0.17		4.67 (0.72)			0.16	
	A32. Comprehensiveness of treatment recommendations	4.53 (0.64)		0.14		4.40 (0.63)			0.14		4.27 (0.88)			0.21	
	A33. Operability of treatment recommendations	4.53 (0.74)		0.16		4.40 (0.74)			0.17		4.27 (0.80)			0.19	
	B11. Interface friendliness	4.47 (0.52)		0.12		4.73 (0.46)			0.10		4.33 (0.62)			0.14	
	B12. Perceived reliability	4.80 (0.56)		0.12		4.53 (0.64)			0.14		4.67 (0.49)			0.10	
	B13. Perceived responsiveness	4.67 (0.49)		0.10		4.73 (0.46)			0.10		4.67 (0.62)			0.13	
	B14. Perceived safety	4.67 (0.49)		0.10		4.27 (0.70)			0.16		4.33 (0.49)			0.11	
	B15. Emotional identification	4.21 (0.70)		0.17		4.43 (0.76)			0.17		4.07 (0.73)			0.18	
	C11. Bias	4.87 (0.35)		0.07		4.40 (0.74)			0.17		4.53 (0.64)			0.14	
	C12. Privacy	4.80 (0.41)		0.09		4.67 (0.62)			0.13		4.53 (0.64)			0.14	
	C13. Hallucination	4.67 (0.49)		0.10		4.07 (0.59)			0.15		4.60 (0.74)			0.16	
	C14. Establishment of ethics committee	4.80 (0.41)		0.09		4.67 (0.72)			0.16		4.20 (0.77)			0.18	
	C21. Presence of a disclaimer notices	4.87 (0.35)		0.07		4.93 (0.26)			0.05		4.27 (0.70)			0.16	
	C22. Generation of factually incorrect information	5.00 (0.00)		0.00		4.67 (0.49)			0.10		4.87 (0.35)			0.07	
	C23. Violation of medical compliance	4.93 (0.26)		0.05		4.87 (0.35)			0.07		4.73 (0.46)			0.10	
	C31. Emergency response mechanisms	4.80 (0.56)		0.12		4.67 (0.49)			0.10		4.67 (0.62)			0.13	
	C32. Access management for minors	4.80 (0.41)		0.09		4.87 (0.35)			0.07		4.60 (0.63)			0.14	
	C33. Presence of privacy terms	4.80 (0.41)		0.09		4.80 (0.41)			0.09		4.40 (0.74)			0.17	

^a^CV: coefficient of variation.

**Table 8:** Final weight coefficients of indicators.

First-, second-, and third-level indices					Weight
**A. Health consultation capability**					0.4112
	**A1. Health information inquiry ability**			0.1272	
		A11. Accuracy in understanding user needs	0.0648		
		A12. Completeness of user information collection	0.021		
		A13. Logical sequence of inquiries	0.0122		
		A14. Recognition ability of multimodal information	0.0219		
		A15. Personalization of communication style	0.0073		
	**A2. Health problem reasoning ability**			0.0821	
		A21. Accuracy of disease risk reasoning	0.0389		
		A22. Consistency of repeated judgments	0.0182		
		A23. Diagnostic ability for complex diseases	0.0094		
		A24. Frequency of medical knowledge updates	0.0056		
		A25. Interpretability of disease reasoning	0.01		
	**A3. Treatment recommendation generation ability**			0.2019	
		A31. Accuracy of treatment recommendations	0.1216		
		A32. Comprehensiveness of treatment recommendations	0.0562		
		A33. Operability of treatment recommendations	0.0241		
**B. User experience**					0.1107
	**B1. Service quality experience**			0.1107	
		B11. Interface friendliness	0.0072		
		B12. Perceived reliability	0.0456		
		B13. Perceived responsiveness	0.0132		
		B14. Perceived safety	0.0336		
		B15. Emotional identification	0.0111		
**C. Ethics and compliance**					0.4781
	**C1. Ethical compliance**			0.1933	
		C11. Bias	0.0539		
		C12. Privacy	0.0491		
		C13. Hallucination	0.0697		
		C14. Establishment of ethics committee	0.0205		
	**C2. Medical compliance**			0.1696	
		C21. Presence of a disclaimer notices	0.0161		
		C22. Generation of factually incorrect information	0.0889		
		C23. Violation of medical compliance	0.0646		
	**C3. Risk prevention mechanism**			0.1152	
		C31. Emergency response mechanisms	0.0605		
		C32. Access management for minors	0.0362		
		C33. Presence of privacy terms	0.0185		

### Weight Determination of Each Indicator

The AHP was used to calculate the weights of each indicator, with higher weights indicating greater importance in evaluating the quality of health conversational AI. In this study, all consistency ratio values of the judgment matrices were automatically adjusted using Yaahp and were found to be less than 0.10, indicating good consistency across all matrices.

The final weight coefficients of all indicators are shown in Table 8. Among the primary indicators, the ranking by weight from highest to lowest was as follows: ethics and compliance (0.4781), health consultation capability (0.4112), and user experience (0.1107).

## Discussion

### Principal Findings

As AI becomes increasingly integrated into health care, HCAI is being used more widely in health communication, clinical decision support, and consultation. Establishing a scientific and systematic quality evaluation framework is therefore essential. Although several evaluation systems have been developed, many still focus primarily on accuracy or clinician-oriented indicators, neglecting critical aspects of real-world product use and comprehensive safety assessment. They pay limited attention to the comprehensive integration of user experience, ethical and compliance considerations, and the robust assessment of multiturn interactions, all of which are essential for HCAI in real consultation settings. Addressing these gaps is critical for improving the quality, safety, and effectiveness of HCAI.

This study employs the Delphi method and AHP to develop an evaluation index system that reflects the perspectives of physicians, users, and regulators, and makes 3 key contributions addressing the limitations of prior work. First, our system is constructed from the perspective of HCAI as an end-user product for daily health consultation, rather than focusing solely on clinical-task performance. Second, it encompasses 3 core, balanced dimensions: health consultation capability, user experience, and ethics and compliance. Third, by using the AHP methodology, the framework supports systematic and operational evaluation through weighted, hierarchical criteria. By addressing these dimensions and the shortcomings of existing frameworks, the proposed system provides a more comprehensive structure that aligns with the real-world needs of HCAI services. The framework provides a theoretical foundation for empirical research, guides practical optimization, and serves as a valuable reference for improving HCAI evaluation standards worldwide.

### Scientific Validity and Rationality of the Evaluation Index System

The final evaluation index system developed in this study comprises 3 primary indicators, 7 secondary indicators, and 28 tertiary indicators. Its structure is comprehensive and well-organized, demonstrating both scientific rigor and practical relevance.

First, the design of the index system was grounded in existing literature, industry standards, and expert consensus. It incorporated both domestic and international approaches to HCAI evaluation, ensuring a solid theoretical foundation and methodological relevance.

Second, the study employed 2 rounds of Delphi expert consultation, combined with AHP for weight assignment, integrating both qualitative judgment and quantitative analysis. Expert opinions showed increasing convergence across rounds, with statistically significant coordination coefficients, indicating a high level of consensus and enhancing the credibility of the evaluation system.

Third, the experts involved in the study were highly representative across disciplines, including health management and policy, medical ethics, computer science, health law, and hospital administration. The panel comprised both academic researchers and experienced practitioners, ensuring a balance of theoretical insight and practical expertise. This interdisciplinary and diverse expert composition enhanced the scientific validity of the evaluation system and conformed to established Delphi methodology standards for expert selection and quality assurance [55,56].

Finally, the experts demonstrated high levels of engagement, authority, and consistency throughout the process, significantly enhancing the reliability of the study’s findings [57]. Both rounds of the Delphi consultation achieved a 100% response rate (16 and 15 experts in rounds 1 and 2, respectively), with a total of 39 modification suggestions proposed, reflecting active participation and strong interest from the expert panel. The Cr values in both rounds exceeded 0.8, confirming the reliability of expert judgments. Additionally, the *P* values of Kendall *W* were all below 0.05, indicating a high level of agreement in weight assessments and providing a solid foundation for the reliability of the constructed index system.

### Analysis of Indicator Weights

The weights of the 3 primary indicators are shown in Table 8: ethics and compliance (0.4781), health consultation capability (0.4112), and user experience (0.1107). These results indicate that ethics and compliance carries the greatest weight in the quality evaluation of HCAI, underscoring its importance as a key dimension for building safe and trustworthy AI systems.

Health consultation capability, which reflects the AI’s professional value and its ability to deliver knowledge-based services, also accounts for a substantial proportion of the overall weight. By contrast, user experience carries a relatively lower weight. This may be because current HCAI products and applications are still in the early stages of development, leading experts to prioritize system safety, compliance, and functionality during the evaluation process, while user interaction is considered a secondary factor.

At present, many HCAI systems face significant risks related to algorithmic bias, data privacy breaches, hallucinations, and lack of medical compliance [58-60]. These risks are closely associated with the ethics and compliance dimension of the evaluation system. Among the tertiary indicators in this study, several related to ethical and safety concerns received relatively high weight values, including “generation of factually incorrect information” (0.0889), “hallucination” (0.0697), “violation of medical compliance” (0.0646), and “emergency response mechanisms” (0.0605). These results indicate that truthfulness, safety, and regulatory compliance of AI-generated content are critical components in assessing the quality of HCAI. In clinical settings, inaccurate recommendations or breaches of patient privacy can have serious consequences and must be addressed as a priority. This finding aligns with concerns raised by other researchers. For instance, some studies have reported that AI in health care may generate hallucinations—that is, inaccurate or fabricated content that could compromise clinical decision-making and patient safety [61]. Wang et al [62] also noted that LLMs may not achieve complete deidentification of training data, thereby raising the risk of exposing sensitive user information.

Within the health consultation capability dimension, the highest weight was assigned to “treatment recommendation generation ability” (0.2019). Among its tertiary indicators, “accuracy of treatment recommendations” (0.1216) and “comprehensiveness of treatment recommendations” (0.0562) were assigned relatively high weights. This indicates strong expert attention to whether AI systems can accurately assess users’ health conditions and provide reliable and helpful advice. These findings are highly consistent with previous studies by Lukac et al [63] and Liu et al [64], both of whom emphasized the importance of AI’s ability to generate dependable treatment recommendations. In addition, the tertiary indicator “accuracy in understanding user needs” (0.0648), under the category of “health information elicitation ability,” and “accuracy of disease reasoning” (0.0389), under “health problem reasoning ability,” also received relatively high weights. This suggests that, at the current stage, experts continue to view AI’s capabilities in understanding user intent and providing accurate reasoning as important considerations.

In comparison, although the user experience dimension received a relatively lower overall weight, certain indicators, such as “perceived reliability” (0.0456) and “perceived safety” (0.0336), still accounted for a meaningful proportion. This reflects the significant impact of users’ perceived trust in AI systems on actual usage experiences. Previous studies have shown that trust is one of the key determinants influencing users’ acceptance and use of conversational AI tools such as ChatGPT [65]. In the health care context, user trust in AI technologies is also considered a critical factor affecting their broader adoption and application [66]. Moreover, some studies have found that, compared with traditional information channels such as online health websites, HCAI systems are better positioned to meet users’ needs for immediacy and convenience during health information-seeking. This is largely due to their ability to engage in real-time interaction and provide personalized responses [67]. These findings further suggest that, in the ongoing optimization of HCAI, user experience should not only be regarded as a key factor influencing technology acceptance but also as a critical lever for enhancing system usability and fostering trust between humans and machines.

### Policy Recommendations

In summary, the development of HCAI remains at an early stage. Ethics and compliance has emerged as the most critical evaluation dimension, while health consultation capability serves as the core functional foundation. Although user experience holds a relatively lower weight, it still plays a significant role in promoting the adoption and practical application of HCAI systems. To further advance the development of HCAI, this study proposes the following policy recommendations:

First, regulatory authorities around the world have imposed increasingly stringent requirements on HCAI-related products. For example, the European Union’s Artificial Intelligence Act [68] mandates a comprehensive regulatory framework for high-risk AI systems, including those used in health care, covering both premarket and postmarket phases [68]. Singapore’s Model AI Governance Framework for Generative AI emphasizes the importance of continuous evaluation and improvement across the entire life cycle of AI systems [69]. Similarly, China’s Interim Measures for the Management of Generative Artificial Intelligence Services clearly stipulate dynamic supervision and safety monitoring of AI products after their deployment [70]. In light of this, it is recommended that the evaluation index system developed in this study be used as the basis for establishing a full–life cycle quality assessment mechanism for HCAI. This mechanism should encompass product development, testing, deployment, and application. Furthermore, efforts should be made to promote its transformation into a widely accepted industry framework through supportive policy initiatives. Such an approach would facilitate the regular evaluation of HCAI products and enable dynamic supervision and continuous quality improvement.

Second, the performance of HCAI systems should be continuously optimized across 3 key dimensions: model capability, ethics and compliance, and user experience. In terms of model capability, targeted training and fine-tuning should be conducted on tasks such as health information elicitation, disease diagnosis, and treatment recommendation to improve the system’s medical professionalism and response accuracy. For ethical and safety considerations, it is necessary to establish a regulatory framework that covers data processing, model outputs, and information usage. This framework should clearly define accountability and responsibility in high-risk scenarios and include mechanisms to prevent misinformation and breaches of privacy. Regarding user experience, improvements should focus on core indicators such as “perceived safety” and “perceived reliability.” This can be achieved by refining language logic, setting boundary warnings, incorporating source attribution, and integrating human-in-the-loop mechanisms. These measures are essential to enhance user understanding of, and trust in, the system.

Finally, HCAI is expected to move beyond the constraints of online consultation platforms and mobile apps. By helping users recognize their own health conditions, understand disease-related knowledge, and enhance early screening, diagnosis, and treatment, HCAI has the potential to improve health literacy and overall well-being. It may thus become a convenient and trustworthy health gatekeeper for the public. HCAI can also be integrated with health care service institutions and medical consortia to support the accuracy and feasibility of hierarchical diagnosis and treatment systems. To promote the effective application of HCAI in diverse scenarios, such as self-assessment and diagnosis, intelligent triage, chronic disease follow-up, and care of older adults, it is necessary to adjust the structure and weight allocation of existing evaluation frameworks. Capability indicators should be refined based on the characteristics of specific tasks, with a particular focus on the model’s adaptability and service effectiveness across different use cases. These efforts will help strengthen the professional performance of HCAI systems in multiple contexts and enhance the overall level of intelligent health care services.

### Strengths and Limitations of Research

Unlike most previous expert consultation studies, this research assessed not only the importance of each indicator but also its sensitivity and feasibility, thereby enhancing the practicality of the constructed evaluation index system [71]. The findings of this study help address a critical gap in the evaluation of HCAI quality. The developed index system can be applied to assess the service quality of existing HCAI systems, identify weaknesses in consultation capability, user experience, ethics and compliance, and provide theoretical support for future product optimization and model iteration. This contributes to the standardized and high-quality development of HCAI. Furthermore, the evaluation system may serve as a reference for industry regulation. It offers a quantitative tool for government agencies, technology platforms, and health care providers to establish standards, identify risks, and improve service quality.

This study has several limitations. First, the expert panel consisted of 15-16 professionals from China. Although the panel was selected to ensure domain expertise, the limited sample size and geographic scope may affect the diversity and generalizability of perspectives. Future research should consider expanding the pool of experts to enhance the authority and generalizability of the conclusions. Second, although this study proposed a structured and multidimensional evaluation framework for HCAI, its practical utility has not yet been validated in real-world settings. While detailed assessment criteria for each third-level indicator are provided in Multimedia Appendix 2, further refinement of operational thresholds and performance benchmarks will be needed to support practical application. Additional empirical validation is required to assess the framework’s applicability and adaptability across different scenarios.

### Conclusions

This study developed a quality evaluation index system for HCAI, consisting of 3 primary indicators, 7 secondary indicators, and 28 tertiary indicators. Expert scoring results indicated that the dimension of “ethics and compliance” had the highest weight, followed by “health consultation capability.” This suggests that, when evaluating the quality of health-related AI systems, priority should be given to information security, medical compliance, and risk management. The study combined the Delphi method with the AHP to ensure the scientific validity of the evaluation indicators and the rationality of their weight distribution. The proposed evaluation framework provides a theoretical reference for the assessment and optimization of HCAI systems.

## Appendix

Multimedia Appendix 1 Expert consultation questionnaire in the first round.

Multimedia Appendix 2 Expert consultation in the second round and analytic hierarchy process questionnaire.

## Abbreviations

AHP: analytic hierarchy process
AI: artificial intelligence
AMIE: Articulate Medical Intelligence Explorer
BLEU: Bilingual Evaluation Understudy
Ca: the basis for expert’s judgment
CPMI: Chinese Patent Medicine Instructions
Cr: expert authority coefficient
CRAFT-MD: the Conversational Reasoning Assessment Framework for Testing in Medicine
Cs: expert familiarity
CV: coefficient of variation
HCAI: health conversational artificial intelligence
LLaMA: Large Language Model from Meta
LLM: large language model
Medical-Diff-VQA: Medical Dataset for Difference Visual Question Answering on Chest X-Ray Images

## Data Availability

The data that support the findings of this study are available from the authors; however, restrictions apply to their availability, as the data were used under license from the experts involved in the Delphi survey for this study and are therefore not publicly available. The data are, however, available from the authors upon reasonable request and with permission from these experts.
